# Study on the impact and clinical effect of high-quality nursing intervention on the quality of life of elderly cataract patients

**DOI:** 10.12669/pjms.40.3.7526

**Published:** 2024

**Authors:** Kunkun Zuo, Zhaoqing Sun, Kai Wen

**Affiliations:** 1 Kunkun Zuo, Ambulatory Surgery Center One, Tianjin Key Laboratory of Retinal Functions and Diseases, Tianjin Branch of National Clinical Research Center for Ocular Disease, Eye Institute and School of Optometry, Tianjin Medical University Eye Hospital, Tianjin, 300384, China; 2 Zhaoqing Sun, Ambulatory Surgery Center One, Tianjin Key Laboratory of Retinal Functions and Diseases, Tianjin Branch of National Clinical Research Center for Ocular Disease, Eye Institute and School of Optometry, Tianjin Medical University Eye Hospital, Tianjin, 300384, China; 3 Kai Wen, Ambulatory Surgery Center One, Tianjin Key Laboratory of Retinal Functions and Diseases, Tianjin Branch of National Clinical Research Center for Ocular Disease, Eye Institute and School of Optometry, Tianjin Medical University Eye Hospital, Tianjin, 300384, China

**Keywords:** High-Quality Nursing Intervention, Cataract Surgery, Quality Of Life, Clinical Effect

## Abstract

**Objective:**

To evaluate the impact and clinical effect of high-quality nursing intervention on the quality of life of elderly cataract patients and its clinical effect.

**Methods:**

This is a clinical comparative study. One hundred and twenty elderly cataract patients admitted to Tianjin Medical University Eye Hospital for surgical treatment were recruited and randomly divided into two groups: the control and experimental group, with 60 cases in each group from January 15, 2021 to January 15, 2022. Patients in the control group were given conventional nursing care in the perioperative period, while those in the experimental group were given high-quality nursing intervention in the perioperative period. The differences in anxiety (SAS) scores, depression (SDS) scores, intraocular pressure (IOP) recovery, the incidence of surgical complications and satisfaction before and after treatment between the two groups were compared and analyzed.

**Results:**

No statistically significant difference was observed between the two groups in physical functioning, psychological functioning, social functioning and material life status scores before intervention(P>0.05). After the intervention, the above indicators improved significantly in the experimental group compared to the control group, with statistically significant differences (P=0.00). Moreover, SAS and SDS decreased significantly in the experimental group compared to the control group, with statistically significant differences(P=0.00).

**Conclusions:**

High-quality nursing intervention improves various benefits in the treatment of elderly cataract patients, such as effectively reducing intraocular pressure, ameliorating patients’ quality of life, lowering the incidence of postoperative complications, and improving patient satisfaction.

## INTRODUCTION

Cataracts, a common clinical ophthalmic disease occurring mostly in middle-aged and elderly patients, have been proven to be positively correlated with age.^1^ In terms of its pathogenesis, the protein of the lens is denatured and cloudy, resulting in blurred vision due to the inability to project objects clearly on the retina.^2^ There are multiple causes of disorders of lens metabolism, including aging of the lens, traumatic irritation, abnormal autoimmune system and so on.^3^ More than 13% of patients with advanced eye disease have cataracts, according to a recent study.^4^ Recent years have witnessed an increasingly serious trend of population aging, and with it, the incidence of cataracts.^5^

Currently, surgery is the preferred means of cataract treatment, of which ultrasonic phacoemulsification of cataract nucleus boasts faster development, less trauma, and favourable treatment effect. It is used as the current main surgical method for the treatment of advanced cataracts.^6^ Elderly patients with cataracts and glaucoma usually have significantly deteriorated body functions and reduced tolerance due to their advanced age. The combined effect of such factors brings higher risk to the surgery. For this reason, effective nursing care should be given in the perioperative period, which is a solid guarantee of successful treatment.^7^ Given the inability of basic ophthalmic nursing to meet the nursing needs of elderly patients, high-quality nursing measures were adopted in this study to provide effective interventions throughout the perioperative period, and its clinical efficacy and safety were evaluated.

## METHODS

This is a clinical comparative study. One hundred and twenty elderly cataract patients admitted to Tianjin Medical University Eye Hospital for surgical treatment were recruited and randomly divided into two groups: the control group and the experimental group, with 60 cases in each group from January 15, 2021 to January 15, 2022. In the experimental group, there were 37 female cases and 23 male cases, aged 64-83 years old, with an average of 72.41±8.64 years old in the control group, there were 38 female cases and 22 male cases, aged 67-83 years old, with an average of 72.57±8.26 years old. No significant difference was observed in the comparison of general data between the two groups, which was comparable (Table-I).

**Table-I:** Comparative analysis of the general data of the experimental group and the control group (*χ̅*±*S*) n=60.

Indicators	Experimental group	Control group	t/ (χ^2^	P
Age (years old)	72.41±8.64	72.57±8.26	0.10	0.92
Female (cases %)	37 (62%)	38 (63%)	0.04	0.85
Medical history (years)	6.73±1.26	6.28±1.43	1.83	0.10
BMI (kg/m^2^)	22.49±5.03	22.62±5.21	0.14	0.89
Comorbidity				
Hyperlipidemia (cases %)	18 (30%)	21 (35%)	0.34	0.56
Diabetes mellitus (%)	22 (37%)	23 (38%)	0.03	0.87
Coronary heart disease (cases %)	6 (10%)	4 (7%)	0.44	0.51

P>0.05.

### Ethical Approval:

The study was approved by the Institutional Ethics Committee of Tianjin Medical University Eye Hospital (No.:2021KY(L)-23, date: August 31, 2021), and written informed consent was obtained from all participants.

### Inclusion criteria:

- Patients meeting the diagnostic criteria for cataracts.^8^
- Patients aged ≥ 60 years old;
- Patients who met the surgical indications of ultrasonic phacoemulsification of cataract nucleus without absolute contraindications to surgery;
- Patients with clear consciousness, no mental disorders, and able to actively cooperate with the implementation of treatment and nursing programs;
- Patients who signed the informed consent form and voluntarily participated in this study;
- Patients with complete clinical data;
- Patients who were able to cooperate with the completion of the study and had good treatment compliance.

### Exclusion criteria:

- Patients with other concurrent ophthalmic diseases;
- Patients with other vital organ dysfunction such as heart, liver and kidney;
- Patients with a severe mental disorder or cognitive dysfunction.

Patients in the control group were given conventional nursing care, i.e., conventional health education upon admission to get familiar with the hospital situation as soon as possible, take medicine as prescribed by the doctor, and receive relevant treatment, conventional preoperative education, prevention and treatment of postoperative complications, etc. They were assessed daily by the responsible nurse for the condition of the operated eyes and were recorded for the occurrence of complications. Common complications after cataract surgery include corneal endothelial edema, elevated intraocular pressure and endophthalmitis. Patients in the experimental group were given high-quality nursing intervention:

***(1) Preoperative nursing:*** Elderly cataract patients were vulnerable to negative emotions such as tension, fear and depression before surgery because of the degeneration of organ functions, poor psychological tolerance, sensitivity and fragility, etc. Before surgery, patients were given psychological counseling by nursing staff to ensure emotional stability and smooth operation. They were informed of the specific items of the preoperative examination and the examination time, the purpose of the examination, and the precautions with the common language used in communication, so as to have a general understanding of the surgery, thus avoiding excessive nervousness affecting the process of the surgery.

***(2) Intraoperative nursing:*** The ward nurses reported the preoperative nursing condition of the patient to the operating room nurse, and gave them a homemade record sheet of the patients’ intraoperative conditions and instructed them to fill it out. The record sheet included detailed records of patients’ intraoperative conditions such as intraoperative vital signs, skin status, and anesthesia recovery status.

***(3) Postoperative nursing:*** After surgery, nurses promptly and comprehensively understood the patients’ surgical condition and assessed their status. For elderly patients who needed to lie in different positions after surgery, the nurses helped them to lie in the correct position, observed and asked them whether they had any discomfort, observed their skin condition, and helped them to move their limbs on time. After surgery, the nurses made regular visits and observed the intraocular conditions of the patients, and cooperated with the doctors to prevent and deal with postoperative complications in time. Considering that patients with sensitive eye sensations may have pain, discomfort and other reactions after surgery, nurses should timely evaluate and deal with the postoperative pain response of patients. For patients with obvious pain, nurses should timely observe the intraocular condition and report and deal with abnormalities in time. They should teach patients to learn relaxation techniques and guide them to correct postoperative activities to avoid aggravating intraocular pain. In addition, they should provide effective psychological care to relieve the physical discomfort caused by stress and anxiety that could aggravate pain manifestations. The two groups of patients were followed up for six months.

### Observation indicators:

All the data was collected by Face to face interview. (1) Comparative analysis of the quality of life of the two groups before and after intervention: The Generic Quality of Life Inventory-74 (GQOLI-74) was used to assess various aspects of patients such as physical function, psychological function, social function, and physical living status. A total of 20 questions and 74 items were set in the questionnaire, each item scored 1-5 points. The higher the score, the higher the quality of life of the patients.^9^ (2) Comparative analysis of anxiety and depression scores: The Self-rating Anxiety Scale (SAS) and Self-rating Depression Scale (SDS)^10^ were employed to assess the emotional change status of the two groups before and after the intervention, respectively, with lower scores indicating better emotional status. (3) The recovery of IOP in both groups after the intervention was examined by taking a conventional non-contact IOP meter. (4) The postoperative complications in both groups were observed, such as corneal edema, bleeding in the anterior chamber of the eye, and exudation in the anterior chamber of the eye. (5) The Patient Satisfaction Questionnaire Short Form (PSQ-18)^11^ was used to compare and analyze patient satisfaction before and after the intervention, including greatly satisfied, more satisfied, satisfied, uncertain, and unsatisfied. Overall satisfaction = (greatly satisfied + more satisfied + satisfied)/total number of cases x 100%.

### Statistical analysis:

All data in this study were statistically analyzed by SPSS 20.0 software, and measurement data were expressed as (*x̅*±S). Two independent sample t-test was used for comparison between groups, paired t test or ANOVA was used to analyze data within groups, and χ^2^ test was used for the comparison of rates. P<0.05 indicates a statistically significant difference.

## RESULTS

No significant difference was observed in the scores of physical functioning, psychological functioning, social functioning and material life status between the two groups before intervention (P>0.05). After the intervention, the above indicators improved significantly in the experimental group compared with the control group, with statistically significant differences (p=0.00) (Table-II).

**Table-II:** Comparative analysis of quality of life scores between the two groups before and after intervention (*χ̅*±*S*) n=60.

Indicators		Experimental group	Control group	t	p
Physical functioning	Before intervention	45.41±5.83	45.82±5.66	0.43	0.58
	After intervention*	56.47±6.28	51.45±6.40	5.47	0.00
Psychological functioning	Before intervention	52.61±6.25	52.74±6.22	0.52	0.43
	After intervention*	58.36±6.31	55.32±6.18	4.37	0.00
Social functioning	Before intervention	54.79±6.21	54.82±6.28	0.31	0.64
	After intervention*	67.92±5.41	63.38±5.39	7.35	0.00
Material life status	Before intervention	46.14±5.39	46.32±6.01	0.58	0.43
	After intervention*	53.18±5.22	48.71±5.41	3.48	0.00

* P<0.05.

No significant difference was observed in the SAS and SDS levels of the two groups before intervention (P>0.05). After the intervention, SAS and SDS decreased significantly in the experimental group compared to the control group, with statistically significant differences (P=0.00) (Table-III). No significant difference was observed in the IOP of both groups before intervention (P=0.70). The IOP was lower in both groups at one month and three months after treatment compared to the pre-treatment period, with statistically significant differences P=0.00). The IOP reduction in the experimental group was more significant than that in the control group (P=0.00), as shown in Table-IV.

**Table-III:** Comparative analysis of the emotional status of the two groups before and after intervention (*χ̅*±*S*) n=60.

Indicators		Experimental group*	Control group	t	p
SAS	Before intervention	60.83±5.41	61.12±5.27	0.30	0.77
	After intervention*	44.81±7.20	48.71±6.23	3.17	0.00
SDS	Before intervention	53.47±5.41	53.69±5.28	0.23	0.82
	After intervention*	42.06±5.53	48.31±6.02	5.92	0.00

* P<0.05.

**Table-IV:** Comparative analysis of IOP recovery between the two groups after intervention (*χ̅*±*S*) n=60.

Group	Before treatment	1 month after treatment*	3 months after treatment*	F	P
Experimental group*	33.87±5.34	24.53±2.76	20.58±2.13	8.78	0.00
Control group*	33.48±5.61	28.72±2.81	25.46±2.20	7.08	0.00
*t*	0.39	8.24	12.34		
*P*	0.70	0.00	0.00		

* P<0.05.

Postoperative complications in both groups were mainly corneal edema, anterior chamber hemorrhage and exudation. The incidence of complications in the experimental group was 3%, lower than that of the control group (15%), with a statistically significant difference (P=0.03) (Table-V). The patient satisfaction rate of the experimental group was 97%, significantly higher than that of the control group (85%), with a statistically significant difference (P=0.04) (Table-VI).

**Table-V:** Comparative analysis of the incidence of postoperative complications between the two groups (*χ̅*±*S*) n=60.

Group	Corneal edema	Anterior chamber hemorrhage	Anterior chamber exudation	Incidence
Experimental group	1	0	1	2 (3%)
Control group	3	2	4	9 (15%)
*χ* ^2^				4.90
*P*				0.03

P<0.05.

**Table-VI:** Comparative analysis of patient satisfaction between the two groups (*χ̅*±*S*) n=60.

Group	Greatly satisfied	More satisfied	Satisfied	Uncertain	Unsatisfied	Overall satisfaction*
Experimental group	34	11	13	1	1	58 (97%)
Control group	30	18	3	6	3	51 (85%)
*χ* ^2^						4.90
*P*						0.03

* P<0.05.

## DISCUSSION

It was confirmed in our study that there was no significant difference in the scores of somatic function, psychological function, social function and physical life status between the two groups before the intervention (P>0.05). After the intervention, the indexes of the experimental group improved significantly compared with the control group, with a statistically significant difference (P=0.00), the incidence of complications was 3% in the experimental group and 15% in the control group, the experimental group was lower than the control group, with a statistically significant difference (P=0.00), with a statistically significant difference (P=0.03).

According to a study^12^, based on patients’ understanding of the disease and treatment, patients can better participate in care and also improve the prevention of the disease and better avoid negative factors.^13^ The preoperative and intraoperative interventions can help patients understand the treatment process, gain their trust and support, and thus actively cooperate with the treatment and care, laying the foundation for the smooth operation and improving the success rate. In this analysis, the anxiety and depression scores of patients in the quality care group were significantly lower than those in the control group (P=0.00). This suggests that quality care measures have a positive effect on reducing negative emotions, while measures such as health guidance strengthen patients’ knowledge and understanding of the disease and increase their awareness of self-care. In addition, the intervention of postoperative nursing measures facilitated the patients’ recovery. Understanding postoperative-related precautions reduce the negative effects caused by wrong behaviors.

The results of this study suggest that the IOP of patients in both groups decreased in one month and three months after treatment compared with that before treatment, and the IOP reduction was more significant in the experimental group than in the control group (P=0.00); 97% of patients in the experimental group were satisfied and 85% of patients in the control group were satisfied. This fully illustrates that the quality nursing measures changed the patients’ bad behavioral habits, improved the patients’ satisfaction and compliance, and accelerated the patients’ recovery. Cataracts, most often found in the elderly population, are caused by local nutritional disorders and the aging of organs. The disease has a significant impact on life, with blurred vision in those who suffer from it, which seriously affects the patient’s life experience.^14^ It is also prone to complications of various ophthalmic diseases, such as astigmatism and glaucoma.^15^ In terms of its pathogenesis, it is a clouding of the lens caused by a variety of reasons. The lens is a vital component of the refractive interstitium, which is colorless and transparent in the normal population, and its transparency is altered to produce visual impairment.^16^

A plurality of factors, such as aging, inflammation, genetics, metabolic abnormalities, toxicity, trauma, and local nutritional disorders, may cause damage to the lens capsule and weaken or lose its barrier function. This, in turn, leads to an increase in its permeability or disruption of lens metabolism, which degenerates lens proteins and forms cloudiness.^17^ Cataracts, the major blindness-causing eye disease in the world, is reported to account for 41% of all blindness.^18^ Their global prevalence is increasing year by year and has seriously affected the quality of life of patients.^19^

Given that current primary care measures in ophthalmology no longer meet the needs, High-quality nursing intervention was adopted in that study. High-quality nursing intervention is an innovative nursing model that assesses patient satisfaction, physician satisfaction, and patient recovery quality indicators, and is a comprehensive nursing intervention model.^20^ By combining the prognostic regression of the disease and the psychological characteristics of the patient, high-quality nursing intervention can take targeted nursing guidance and ensure that the patient maintains a physical, psychological and socially pleasant state during treatment through continuous care. At the same time, continuous nursing after discharge can reduce adverse emotions and is a scientific extension of in-hospital nursing.^21^ Preoperative psychological interventions, intraoperative interventions, health education, postoperative care, and discharge instructions are used to improve patients’ negative emotions, thereby improving treatment outcomes and facilitating postoperative recovery.

### Limitations:

It inclues a small number of patients were included and the follow-up time was short, and the study was homogeneous because only elderly patients were involved. In future, the sample size will be further expanded, the follow-up time will be extended, so as to make the study content more perfect.

## CONCLUSIONS

To put it in a nutshell, high-quality nursing intervention boasts various benefits in the treatment of elderly cataract patients in the perioperative period, such as promoting the recovery of vision, reducing complications, and ameliorating the patient’s poor psychological status. It improves the quality of nursing and satisfaction in general and is worth popularizing in the clinical setting.

### Authors’ Contributions:

**KZ:** Carried out the studies, collection of data, and drafted the manuscript, are responsible and accountable for the accuracy and integrity of the work.

**ZS:** Performed the statistical analysis and participated in its design.

**KW:** Participated in acquisition, analysis, or interpretation of data, drafting the manuscript.

All authors read and approved the final manuscript.
